# Supplementary material for: Pericentromeric hypomethylation elicits an interferon response in an animal model of ICF syndrome
Source: eLife. 2018 Nov 28;7:e39658. doi: 10.7554/eLife.39658 (PMC6261255; doi:10.7554/eLife.39658)
Supplement: Supplementary file 1. [file elife-39658-supp1.docx]

**Supplementary File 1: List of mutant alleles**

| Name of allele | Mutation in | Reference | Mutati-on | Genotyping Primers | Restriction  Enzyme for genotyping |
| --- | --- | --- | --- | --- | --- |
| *mk22* | *zbtb24* | This publication | ~8kb large deletion | F (P1): AGTCCTCGCTCTGCACTCAG  R (P3): CTCTTGGCGGTGAAACACTT | N/A |
| *mk19* | *zbtb24* | This publication | 8bp deletion | F (P1): AGTCCTCGCTCTGCACTCAG  R (P2): TCTCGTCCACCAACACGAC | Fnu4HI |
| *mk28* | *mavs* | This publication | 4bp deletion | F: ACAGCAGGTGGAGCAAGTTT  R: TGAGGTGGAGATGGGAGATT | Fnu4HI |
| *mk29* | *mda5* | This publication | 7bp deletion | F: GCTCACTGGAGGATCACCAT  R: ACCACCTTGTTGACCAGGAC | BsaJI |
| *mk30* | *tmem173 (STING)* | This publication | 10bp deletion | F: TTTCTGTGTGGCTCTGTCAAGT  R: AGCGATAATTCCAGCTCTTTCA | BssHII |
| *hu3568* | *myd88* | van der Vaart et al., 2013 | Point mutation | F: GAGGCGATTCCAGTAACAGC  R: GAAGCGAACAAAGAAAAGCAA | MseI |
| *s904* | *dnmt1* | Anderson et al., 2009 | 1bp frame-shift | Genotyped based on GFP labelling as described in Goll et al., 2009. | N/A |
