# Supplementary material for: Pericentromeric hypomethylation elicits an interferon response in an animal model of ICF syndrome
Source: eLife. 2018 Nov 28;7:e39658. doi: 10.7554/eLife.39658 (PMC6261255; doi:10.7554/eLife.39658)
Supplement: Supplementary file 2. [file elife-39658-supp2.docx]

**Supplementary File 2. List of primers**

**qPCR**

| **Name** | **Sequence** |
| --- | --- |
| q_zbtb24_F | TCTGCACTCAGCAACACACA |
| q_zbtb24_R | CGGTGAACAATGCTGAGAAA |
| q­_IgZ_F | AAAGCAACGATACCAAAGTG (Page et al., 2013) |
| q_IgZ_R | AACAGCTTGCAAGACAATTC (Page et al., 2013) |
| q_IgD_F | GACACATTAGCCCATCAGCA (Page et al., 2013) |
| q_IgD_R | CTGGAGAGCAGCAAAAGGAT (Page et al., 2013) |
| q_IgM_F | GAAGCCTCCAATTCTGTTGG (Zimmerman et al., 2011) |
| q_IgM_R | CCGGGCTAAACACATGAAG (Zimmerman et al., 2011) |
| q_stat1b_F | GCTGCTCCAATGAAGGTTCC (Li et al., 2014) |
| q­­_stat1b_R | GCAACGGGTCTTGCAACAG (Li et al., 2014) |
| q_irf7_F | AAAGTGGGCAGTACGAAGGT |
| q_irf7_R | TCCATTTTGCTTTGTCGTTAG |
| q_irf1b_F | CGGATGAAGAGTCTGTCTCA (Briolat et al., 2014) |
| q_irf1b_R | TGCCGAGTGAGCTCTAAGAT (Briolat et al., 2014) |
| q_mxa_F | CTGCTTTTCCCAGAACTTCG |
| q_mxa_R | CCCCGGTACTTGACTTCGTA |
| q_ifi27_F | CTACTTACAGCCGCCGTAGC |
| q_ifi27_R | GCAGCTGACATCATTGAGGA |
| q_ ifi44l_F | CAAGCTGGAGCAGGAAAGTC |
| q_ ifi44l_R | ACCAGGGGCAAAGTTTTCTT |
| q_ lgals9_F | GGGCAGAAGAAAAACATGGA |
| q_ lgals9_R | CAGTGAAGCGGTGCTTGTAA |
| q_gbp1_F | CCGCAGTGAGAAAGAAGGAC |
| q_gbp1_R | TGATCCGTTCACATTCTCCA |
| q_CRP_F | GGCTCAATCCAAAAGGAACA |
| q_CRP_R | ATGACCTTCTCCCTCAAGCA |
| q_ il22ra2_F | CTACCCTCAGTGGGAAACCA |
| q_ il22ra2_R | TCAGTGGAGTTCTCGGAGGT |
| q_ il1b_F | GACTTCGCAGCACAAAATGA |
| q_ il1b_R | CACTTCACGCTCTTGGATGA |
| q_CCL20_F | ATATGGGCCGCTGAACTATG |
| q_CCL20_R | GGCATCTATACGGCACACCT |
| q_caspbl_F | CCTCGAGTCGAAACTTCTGG |
| q_caspbl_R | ATGATCCCTCGTGGTCTCTG |
| q_tnfa_F | CAGGGCAATCAACAAGATGG (Marjoram et al., 2015) |
| q_tnfa_R | TGGTCCTGGTCATCTCTCCA (Marjoram et al., 2015) |
| q_bactin1_F | CGAGCAGGAGATGGGAACC (McCurley and Callard, 2008) |
| q_bactin1_R | CAACGGAAACGCTCATTGC (McCurley and Callard, 2008) |
| q_18S_F | TCGCTAGTTGGCATCGTTTATG (McCurley and Callard, 2008) |
| q_18S_R | CGGAGGTTCGAAGACGATCA (McCurley and Callard, 2008) |
| q_Sat1_F | GTCTCTGACTGAGTTTGCATTAC |
| q_Sat1_R | ACATTCTGAATTGGACGTTGA |
| q_Sat1_ChIP_F | AAGCAAGTTGCAAGTGAAAATCT |
| q_Sat1_ChIP_F | AGTCAGCCAGCAGAGAGGTC |
| q_ERV1-1_F | GTGTTCCGGAGAAAGTGGAA |
| q_ERV1-1_R | ACCCTCGTGCAGTGGTTTAG |
| q_ERV1-3_F | ATCACTATCCCGTGGCTGAG |
| q_ERV1-3_R | ATGTCCTCCACTCGCTTGAG |
| q_LTR-2_F | GGTGTCGTTAGAATGCCCTTGAC (Houwing et al., 2008) |
| q_LTR-2_R | GGTTATACCTGTGGGTCACGTG (Houwing et al., 2008) |
| q_ZFERV1_F | CAAAACTGGGGTTTGGAAGA |
| q_ZFERV1_R | CCCTGCTCCATTGTCTCAGT |
| q_BEL20_F | GTGTCACTTCCCCAAGTCGT |
| q_BEL20_R | GAAAGTGCCTCCAGAAGTGC |
| q_GypsyDR-2_F | GAAATCACCTGTGCATTTAC (Houwing et al., 2008) |
| q_GypsyDR-2_R | ATGCAGACATTGGGTAAAGC (Houwing et al., 2008) |
| q_Dirs1a_F | GGGTGCGTCACGCTTGC (Houwing et al., 2008) |
| q_Dirs1a_R | GTAACCTCGAACGTTCCCC (Houwing et al., 2008) |
| q_L1-1_F | AAATGCTTGGACATGGAAGG |
| q_L1-1_R | TCTGCTGCATCTTGGAACTG |
| q_L1-5_F | GCACAAAGGACAAATTCACTGGAC (Houwing et al., 2008) |
| q_L1-5_R | GTCCACGTTTAGTATTACAGTTGC (Houwing et al., 2008) |
| q_Ngaro_F | GGAGCGATCGAGACCTACC (Houwing et al., 2008) |
| q_Ngaro_R | CAATCATATCACGTGCTCCTCTCG (Houwing et al., 2008) |
| q_EnSpmN1_F | GATTGGCCATTGTGTTCACATGC (Houwing et al., 2008) |
| q_EnSpmN1_R | GCTGTGACTGTCATAGGTTTACC (Houwing et al., 2008) |
| q_Polinton_F | CCTGACAATGTTGTCAGCCTG (Houwing et al., 2008) |
| q_Polinton_R | CATGAAAGCTAAGGGTATAACTCTG (Houwing et al., 2008) |

**Primers for probe synthesis for southern blotting**

| **Name** | **Sequence** |
| --- | --- |
| Sat1_F | TGTTTTAGACAACATTTCATGCAC |
| Sat1_R | AGTCAGCCAGCAGAGAGGTC |
| DANA_F | GGCGACGCAGTGGCGCAGTGGG (Anderson et al., 2009) |
| DANA_R | TTTTCTTTTTGGCTTAGTCCC (Anderson et al., 2009) |
| SINE_HE1_F | TGGCTCAGTGGTTAGCACTG |
| SINE_HE1_R | TTTATCAGGGGTCGCCACAG |
| L1-10_F | ATGGAAGAGGAAGGCAAGGT |
| L1-10_R | GCAGGCCAATTAGTCTCAGG |
| Kolobok_F | AGTGAAGCACAGTTGAGCGA |
| Kolobok_R | ACACTCCTGGATCAGTCGGA |
| ERV1-3-LTR_F | TAAACATGTGTAGTGGAAACTTACAGC |
| ERV1-3-LTR_R | TAAAGAGGCGCTCTCTGTGGT |
| ERV4-DR1_F | GTGGAAAACAGGGTCATTGG |
| ERV4-DR1_R | CACGAAGGCATAAATGCAAA |
| Gypsy21_LTR_F | TTTAAATCTTAAAATTGCCTAAAGGT |
| Gypsy21_LTR_R | TTGCACGCGATGACAACC |
| ZFERV2-LTR_F | TGTCCCATAGGGTGTTGGTT |
| ZFERV2-LTR_R | CAGTGCATGTCCAAAAATGG |

**References:**

Anderson, R.M., Bosch, J.A., Goll, M.G., Hesselson, D., Dong, P.D., Shin, D., Chi, N.C., Shin, C.H., Schlegel, A., Halpern, M.*, et al.* (2009). Loss of Dnmt1 catalytic activity reveals multiple roles for DNA methylation during pancreas development and regeneration. Dev Biol *334*, 213-223.

Briolat, V., Jouneau, L., Carvalho, R., Palha, N., Langevin, C., Herbomel, P., Schwartz, O., Spaink, H.P., Levraud, J.P., and Boudinot, P. (2014). Contrasted innate responses to two viruses in zebrafish: insights into the ancestral repertoire of vertebrate IFN-stimulated genes. J Immunol *192*, 4328-4341.

Houwing, S., Berezikov, E., and Ketting, R.F. (2008). Zili is required for germ cell differentiation and meiosis in zebrafish. EMBO J *27*, 2702-2711.

Li, Y., Esain, V., Teng, L., Xu, J., Kwan, W., Frost, I.M., Yzaguirre, A.D., Cai, X., Cortes, M., Maijenburg, M.W.*, et al.* (2014). Inflammatory signaling regulates embryonic hematopoietic stem and progenitor cell production. Genes Dev *28*, 2597-2612.

Marjoram, L., Alvers, A., Deerhake, M.E., Bagwell, J., Mankiewicz, J., Cocchiaro, J.L., Beerman, R.W., Willer, J., Sumigray, K.D., Katsanis, N.*, et al.* (2015). Epigenetic control of intestinal barrier function and inflammation in zebrafish. Proc Natl Acad Sci U S A *112*, 2770-2775.

McCurley, A.T., and Callard, G.V. (2008). Characterization of housekeeping genes in zebrafish: male-female differences and effects of tissue type, developmental stage and chemical treatment. BMC Mol Biol *9*, 102.

Page, D.M., Wittamer, V., Bertrand, J.Y., Lewis, K.L., Pratt, D.N., Delgado, N., Schale, S.E., McGue, C., Jacobsen, B.H., Doty, A.*, et al.* (2013). An evolutionarily conserved program of B-cell development and activation in zebrafish. Blood *122*, e1-11.

Zimmerman, A.M., Moustafa, F.M., Romanowski, K.E., and Steiner, L.A. (2011). Zebrafish immunoglobulin IgD: unusual exon usage and quantitative expression profiles with IgM and IgZ/T heavy chain isotypes. Mol Immunol *48*, 2220-2223.
