# Supplementary material for: Pericentromeric hypomethylation elicits an interferon response in an animal model of ICF syndrome
Source: eLife. 2018 Nov 28;7:e39658. doi: 10.7554/eLife.39658 (PMC6261255; doi:10.7554/eLife.39658)
Supplement: Supplementary file 3. [file elife-39658-supp3.docx]

**Supplemental File 3: List of Oligos (5’-----3’)**

**Oligos for mutagenesis**

| **Name** | **Sequence** |
| --- | --- |
| mavs_cc_F (oligo based) | GAAATTAATACGACTCACTATAGGACATGTCAGGAGCTGCTTGTTTTAGAGCTAGAAAT |
| mda5_cc_F (oligo based) | GAAATTAATACGACTCACTATAGGTGATAAACACTGCGACCCGTTTTAGAGCTAGAAAT |
| Invariant reverse oligo | AAAAGCACCGACTCGGTGCCACTTTTTCAAGTTGATAACGGACTAGCCTTATTTTAACTTGCTATTTCTAGCTCTAAAAC |
| STING_cc_F (cloning based) | TAGGCAGCCTGCTGCGCGCTCT |
| STING_cc_R (cloning based) | AAACAGAGCGCGCAGCAGGCTG |

gRNA target sequence is underlined

**Oligos for in vitro transcription of Sat1**

| **Name** | **Sequence** |
| --- | --- |
| Sat1-FT3 | TTGAAATTAACCCTCACTAAAGGGAGACTGGTTTTATTACATTCTGAATTGG |
| Sat1-RT7 | TTGATAATACGACTCACTATAGGGAGATCCAGCCATAAAATGCATCA |
| b-actin-FT3 | TTGAAATTAACCCTCACTAAAGGGAGAACTTTGAGCTCCTCCACACG |
| b-actin-RT7 | TTGATAATACGACTCACTATAGGGAGAACTCGGTGATGACGTTCTCG |
| GFP-FT3 | TTGAAATTAACCCTCACTAAAGGGAGATATATCATGGCCGACAAGCA |
| GFP-RT7 | TTGATAATACGACTCACTATAGGGAGAGAACTCCAGCAGGACCATGT |

**Oligos for TASA-TD**

| **Name** | **Sequence** |
| --- | --- |
| b-actin sense TAG | GCACACGACGACAGACGACGCACCAACGGAAACGCTCATTGC |
| b-actin anti-sense TAG | GCACACGACGACAGACGACGCACCGAGCAGGAGATGGGAACC |
| sat1 sense TAG | GCACACGACGACAGACGACGCACACATTCTGAATTGGACGTTGA |
| sat1 antisense TAG | GCACACGACGACAGACGACGCACGTCTCTGACTGAGTTTGCATTAC |
| b-actin sense | CGAGCAGGAGATGGGAACC |
| b-actin antisense | CAACGGAAACGCTCATTGC |
| sat1 sense | GTCTCTGACTGAGTTTGCATTAC |
| sat1 antisense | ACATTCTGAATTGGACGTTGA |
| TAG primer | GCACACGACGACAGACGACGCAC |
